# NKTR-102 Efficacy versus irinotecan in a mouse model of brain metastases of breast cancer

**DOI:** 10.1186/s12885-015-1672-4

**Published:** 2015-10-13

**Authors:** Chris E. Adkins, Mohamed I. Nounou, Tanvirul Hye, Afroz S. Mohammad, Tori Terrell-Hall, Neel K. Mohan, Michael A. Eldon, Ute Hoch, Paul R. Lockman

**Affiliations:** 1 Department of Basic Pharmaceutical Sciences, West Virginia University Health Sciences Center, 1 Medical Center Drive, Morgantown, WV 26506-905 USA; 2 School of Pharmacy, Department of Pharmaceutical Sciences, Texas Tech University Health Sciences Center, Amarillo, TX 79106 USA; 3 Faculty of Pharmacy, Department of Pharmaceutics, Alexandria University, Alexandria, Egypt; 4 Nektar Therapeutics, San Francisco, CA 94158 USA

**Keywords:** Breast cancer, Brain metastasis, PEGylated irinotecan, NKTR-102

## Abstract

**Background:**

Brain metastases are an increasing problem in women with invasive breast cancer. Strategies designed to treat brain metastases of breast cancer, particularly chemotherapeutics such as irinotecan, demonstrate limited efficacy. Conventional irinotecan distributes poorly to brain metastases; therefore, NKTR-102, a PEGylated irinotecan conjugate should enhance irinotecan and its active metabolite SN38 exposure in brain metastases leading to brain tumor cytotoxicity.

**Methods:**

Female nude mice were intracranially or intracardially implanted with human brain seeking breast cancer cells (MDA-MB-231Br) and dosed with irinotecan or NKTR-102 to determine plasma and tumor pharmacokinetics of irinotecan and SN38. Tumor burden and survival were evaluated in mice treated with vehicle, irinotecan (50 mg/kg), or NKTR-102 low and high doses (10 mg/kg, 50 mg/kg respectively).

**Results:**

NKTR-102 penetrates the blood-tumor barrier and distributes to brain metastases. NKTR-102 increased and prolonged SN38 exposure (>20 ng/g for 168 h) versus conventional irinotecan (>1 ng/g for 4 h). Treatment with NKTR-102 extended survival time (from 35 days to 74 days) and increased overall survival for NKTR-102 low dose (30 % mice) and NKTR-102 high dose (50 % mice). Tumor burden decreased (37 % with 10 mg/kg NKTR-102 and 96 % with 50 mg/kg) and lesion sizes decreased (33 % with 10 mg/kg NKTR-102 and 83 % with 50 mg/kg NKTR-102) compared to conventional irinotecan treated animals.

**Conclusions:**

Elevated and prolonged tumor SN38 exposure after NKTR-102 administration appears responsible for increased survival in this model of breast cancer brain metastasis. Further, SN38 concentrations observed in this study are clinically achieved with 145 mg/m^2^ NKTR-102, such as those used in the BEACON trial, underlining translational relevance of these results.

**Electronic supplementary material:**

The online version of this article (doi:10.1186/s12885-015-1672-4) contains supplementary material, which is available to authorized users.

## Background

The overall survival rates for many cancers have not changed over the last few decades, with the exception of certain subtypes of cancer [1–4]. The incidence of brain metastases (BM) continues to increase [5] with current estimates suggesting approximately 600,000 people in the U.S. suffer from some brain malignancy. Brain tumors rank second among causes of cancer-related deaths in individuals under the age of 20, and the fifth leading cause of cancer-related deaths in females aged 20–39 [5]. Brain metastases are the predominant form of brain malignancies, in which 20-40 % of adults with different types of cancers eventually develop brain metastases [6–10]. Breast cancer represents the second most common source of brain metastases [11]; moreover, the incidence of brain metastases of breast cancer (BMBC) in HER2^+^ and triple negative breast cancer (TNBC) is approximately 35 % [12]. Current therapeutic options in treating TNBC brain metastases such as surgery, whole brain radiotherapy, stereotactic radiosurgery, and chemotherapy fail in providing significant progress in treating brain metastases [11, 13] and are mostly palliative [14].

A major obstacle for effective chemotherapeutic activity against BM is drug penetration across the blood–brain barrier (BBB) and the blood-tumor barrier (BTB). The BBB serves as a protective interface that sequesters the brain from undesired chemicals by utilizing physical barriers, efflux transporters, and enzymatic degradation. Together, these components functionally regulate brain penetration of numerous small and large molecules such as anticancer drugs [15]; it is estimated that less than 2 % of drugs targeting the CNS enter clinical trials because of inefficient distribution into brain [16]. The vasculature associated with brain metastases (BTB) becomes compromised resulting in elevated permeability compared to normal BBB; however, the extent of BBB opening following its disruption by the formation of brain metastasis is limited, preventing small molecule chemotherapeutics to reach efficacious levels in the majority of metastatic lesions [17].

**Fig. 1:**
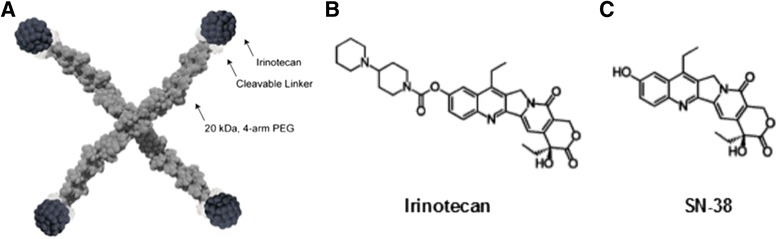
Structures of NKTR-102 (**a**), irinotecan (**b**), and the active metabolite SN38 (**c**)

The application of nanotechnology and polymer chemistry shows promise in animal models of CNS tumors, in particular, glioblastoma multiforme [18–20]. Several drugs applying nanotechnology or polymer chemistry are currently in clinical development for CNS tumors, including ANG1005, in which paclitaxel is conjugated to a peptide vector [18, 21], 2B3-101, a glutathione-PEGylated doxorubicin [22, 23], and MM-398, a liposomal encapsulation of CPT-11 [24]. NKTR-102 (Etirinotecan pegol) is a long-acting polymer conjugate of irinotecan designed to provide continuous exposure of SN38 in tumors while avoiding high irinotecan and SN38 C_max_, which is associated with unwanted side effects [25]. A member of the camptothecin class of topoisomerase 1 (Top1) inhibitors, irinotecan (Camptothecin-11; CPT-11), is a widely used chemotherapeutic agent [11]. CPT-11 is indicated for the treatment of colorectal cancer in combination with 5-fluorouracil (5-FU) and folinic acid (first line) and as a single agent in patients with disease progression following initial 5-FU-based therapy (second line) [26–28]. Top1 inhibition with irinotecan has shown clinical benefit in a wide variety of tumors, including central nervous system cancers [29–31]. In its idealized form, NKTR-102 consists of a 4-arm PEG polymer with a nominal molecular weight of 20 kDa, a hydrolysable ester-based linker, and one irinotecan molecule at the end of each arm (Fig. 1). Upon administration, the linker slowly hydrolyzes resulting in sustained exposure to irinotecan that is subsequently metabolized to the active metabolite SN38 (Fig. 1) [32–35]. NKTR-102 exhibits improved drug penetration into tumors resulting in improved efficacy over irinotecan in a variety of mouse models of human cancers [36], improved peripheral pharmacokinetics [37], and promising clinical activity in metastatic ovarian [38] and breast cancers [39].

We, hypothesized that PEGylation of irinotecan would result in elevated and sustained SN38 concentrations in brain metastases of breast cancer by 1) enhancing passive diffusion of the conjugate from blood into brain via the epithelial tight junction dysregulation at the BTB and 2) bypassing various BBB and BTB efflux transporters, such as P-glycoprotein, that function to restrict drug uptake into brain and brain metastases [40, 41], and 3) releasing SN38 intracellulary within brain metastases at concentrations that result in tumor cell cytotoxicity.

Here, we present encouraging survival and pharmacokinetic (PK) results for NKTR-102 in an experimental mouse model of TNBC brain metastasis. NKTR-102 crosses the BTB, accumulates in brain tumor tissue and serves as a reservoir for release of SN38. The tumor/plasma ratios of SN38 after irinotecan and NKTR-102 administration were 2.8 and 31 respectively. Furthermore, the tumor/plasma ratio of NKTR-102 was 170 compared to 4 for irinotecan. Equally important, tumor SN38 concentrations after NKTR-102 is greater than 20 ng/mL for 168-h, while tumor SN38 concentrations after irinotecan administration only exceeded 1 ng/mL for up to 2-h. This preferential targeting of CNS tumors results in regression of brain metastases and prolongs mouse survival. Plasma SN38 trough concentrations observed in this model are achieved clinically with 145 mg/m^2^ NKTR-102, which is a dose used in the Phase 3 BEACON study in patients with metastatic breast cancer, thereby emphasizing the potential translational relevance of these results.

## Methods

### Chemicals

Irinotecan, radiolabeled [^14^C]-irinotecan, NKTR-102 (PEGylated irinotecan, etirinotecan pegol), and [^14^C]-NKTR-102 were supplied by Nektar Therapeutics (San Francisco, CA). All other chemicals were of analytical grade and were purchased from Sigma-Aldrich (St. Louis, MO).

### Animals

Female athymic nude mice (Charles River Laboratories, Kingston, NY) were used for all experiments in this study. Mice were housed in microisolator cages with a 12-h light/dark cycle and received sterilized food and water *ad libitum*. All animal work was approved by Texas Tech University Health Sciences Center’s Institutional Animal Care and Use Committee (IACUC protocols 06024 & 06026) and West Virginia University’s Animal Care and Use Committee (ACUC protocol 13–1207). All animal work followed internationally recognized guidelines. Human ethics approval for this study is not applicable because no human subjects were involved in this study.

### Cell culture

Brain-seeking human metastatic breast cancer cells stably transfected to express firefly luciferase (MDA-MB-231Br-Luc) were kindly provided by Dr. Patricia Steeg, National Institutes of Health (NIH), Center for Cancer Research. MDA-MB-231Br-Luc cells were cultured in Dulbecco's Modified Eagle's medium (DMEM) supplemented with 10 % fetal bovine serum (FBS). Only cells in passages 2–10 were used. All cells were cultured at 37 °C with 5 % CO_2_.

### Uptake of irinotecan and NKTR-102 in brain tumors

Human MDA-MB-231Br-Luc cells (5 × 10^5^) were implanted intracranially as previously described [42]. Tumors were allowed to grow (30 days or until neurological symptoms developed) prior to intravenous administration of irinotecan (50 mg/kg) or NKTR-102 (50 mg/kg). Animals (*n* = 5/timepoint) were sacrificed under anesthesia (ketamine/xylazine; 100 mg/kg and 8 mg/kg respectively) at pre-determined time points (pre-dose, 2, 6, and 24-h after irinotecan; pre-dose, 6, 24, 168-h after NKTR-102) to collect blood and tumor samples. Plasma and brain tumor samples were assayed for NKTR-102, irinotecan, and SN38 using liquid chromatography–tandem mass spectrometry (LC/MS/MS).

### Uptake of irinotecan and NKTR-102 in brain and brain metastases

Anesthetized (isoflurane) animals were inoculated with MDA-MB-231Br-Luc cells (1.75 × 10^5^) in the left cardiac ventricle consistent with previous methodology [43]. Approximately 40 days after intracardiac injection, 2 mice per sampling time (2-h for irinotecan and 6-h for NKTR-102) received intravenous injections of 50 mg/kg ^14^C-NKTR-102 or ^14^C-irinotecan (4 μCi). Brains were removed, sectioned, and mounted onto slides for quantitation of radioactivity in BM and BDT using quantitative autoradiography (QAR)*.*

### Survival of animals bearing established brain metastases after treatment

Animals were injected intracardially with MDA-MD-231Br-Luc as described above followed by whole body bioluminescence imaging (BLI) to confirm successful injections. Metastases were allowed to develop for 21 days. On day 21, treatment with vehicle (6 mg/mL lactic acid in 5 % dextrose in H_2_O, pH 5–6, *n* = 18), irinotecan (50 mg/kg, *n* = 10), and NKTR-102 (10 or 50 mg/kg, *n* = 10) was initiated via tail vein injection and repeated once weekly along with bioluminescence imaging. Animals were sacrificed under anesthesia (as described above) once neurological symptoms became noticeable. Brains from select animals (*n* = 4/group) were harvested, sectioned, slide mounted, and stained with hematoxylin and eosin (H&E) to visualize brain metastases. The size and number of brain metastases were evaluated using an Olympus MVX10 microscope with a 2X objective (NA = 0.5). Bioluminescence images were acquired 15 min after a intraperitoneal injection of D-luciferin potassium salt (150 mg/kg; PerkinElmer, Waltham, MA) using an IVIS Lumineer XV (PerkinElmer). To confirm successful injection and generation of reproducible large brain metastasis, animals were imaged 24 and 48-h post intracardiac injection. Tumor growth was monitored via BLI before the start of treatment and twice weekly thereafter. Regions of interest (ROIs) were drawn according to the circumference of the cranium and all data were reported as radiance (photons/s/cm^2^/steradian).

### Data analysis

Tumor burden (the number of metastases) and sizes were each compared statistically across treatments using one-way ANOVA followed by Bonferroni’s multiple comparison correction. Differences between treatments were considered statistically significant at *p* < 0.05. Data are reported as Mean ± Standard Error of Mean (SEM) (GraphPad^®^ Prism 5.0, San Diego, CA). Animal survival was used as an additional measure of treatment efficacy. See Additional file 1 for details regarding chemical reagents, cell culture, LC/MS/MS, quantitative autoradiography, and histology.

## Results

### NKTR-102 crosses the BTB, accumulates in brain tumor tissue and serves as reservoir for release of SN38

**Fig. 2:**
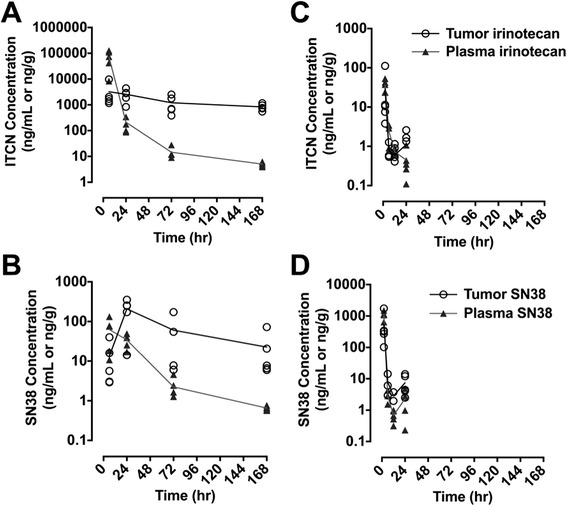
Plasma and tumor concentration-time profiles of irinotecan and active metabolite SN38 after IV bolus administration of NKTR-102 (**a** and **b**) or irinotecan (**c** and **d**) to NU/NU mice with established, orthotopic MDA-MB-231Br brain tumors. Symbols represent individual concentrations, solid line represents mean concentrations (*n* = 5 per time point)

In our first set of experiments, we set out to determine the plasma and brain tumor concentrations of NKTR-102, irinotecan, and their active metabolite SN38 after intravenous administration of either irinotecan or NKTR-102 to mice bearing intracranially implanted tumors. Plasma and brain tumor concentration time profiles of irinotecan, NKTR-102 and SN38 differed significantly between irinotecan and NKTR-102 treatments (Fig. 2). After conventional irinotecan administration, highest concentrations of both irinotecan and SN38 were observed at 2 h (Fig. 2c and d). Both analytes essentially cleared from circulation within 12 h, consistent with previous reports [44]. Tumor irinotecan and SN38 concentrations generally followed kinetics of both entities in plasma and declined 80-fold and 30-fold from their respective tumor C_max_ values 24 h after dosing (Table 1). Brain tumor to plasma concentration ratios after irinotecan administration ranged between 0.5 and 4 for irinotecan and 0.8-2.8 for SN38 during the 24-h sampling period.

**Table 1:** Plasma and Brain Tumor Concentrations after Administration of Irinotecan or NKTR-102

Treatment	NKTR-102				Conventional Irinotecan			
Time (hr)	6	24	72	168	2	6	12	24
	Irinotecan Equivalent Concentration ± SEM (ng/mL or ng/g)				Irinotecan Concentration ± SEM (ng/mL or ng/g)			
Plasma	72450 ± 48790	210 ± 126	14 ± 7.5	5.0 ± 0.09	1100 ± 306	2.9 ± 1.4	0.7 ± 0.3	2.3 ± 1.8
Tumor	3200 ± 3700	2572 ± 1323	1207 ± 904	833 ± 240	554 ± 667	7.7 ± 5.8	2.8 ± 1.2	7.4 ± 5.2
Tumor/Plasma	0.4	12	80	170	0.5	2.7	3	4
SN38 Concentration ± SEM (ng/mL or ng/g)								
Plasma	63 ± 50	34 ± 13	2.2 ± 1.4	0.65 ± 0.08	36 ± 12	2.4 ± 1.3	0.7 ± 0.1	0.4 ± 0.4
Tumor	13 ± 16	208 ± 126	60 ± 78	23 ± 28	29 ± 46	0.8 ± 0.4	0.6 ± 0.4	1.1 ± 1.1
Tumor/Plasma	0.2	6	27	31	0.8	0.3	0.9	2.8

Plasma and brain tumor concentrations for parent drug and active metabolite SN38 after a 50 mg/kg IV bolus injection of either NKTR-102 or irinotecan to NU/NU mice with established, orthotopic MDA-MB-231Br brain tumors. Results are expressed as mean ± SEM (*N* = 5 per time point)

After administration of NKTR-102, plasma NKTR-102 and SN38 concentrations were detectable through the 168 h sampling period (Fig. 2a and b). Brain tumor NKTR-102 concentrations continued to accumulate, eventually exceeding corresponding plasma concentrations by 170-fold 168-h after dosing (Table 1). Unlike administration of conventional irinotecan, brain tumor NKTR-102 concentrations declined by 4-fold compared to corresponding C_max_ value by 168-h post dose. Similarly, SN38 concentrations accumulated in brain tumor, reaching a C_max_ at 24-h after dosing and exceeded plasma concentrations by 30-fold. 22Tumor SN38 concentrations following NKTR-102 administration exceeded 200-fold 24-h post dose compared to irinotecan. Equally important, tumor SN38 concentrations after NKTR-102 were maintained at greater than 20 ng/mL for 168-h, compared to 1 ng/mL for up to 4-h after dosing with conventional irinotecan. Hence, administration of NKTR-102 maintained therapeutic SN38 concentrations [33] for nearly 7 days, compared to fewer than 4-h following irinotecan administration.

### NKTR-102 leads to high concentrations in brain metastases

**Fig. 3:**
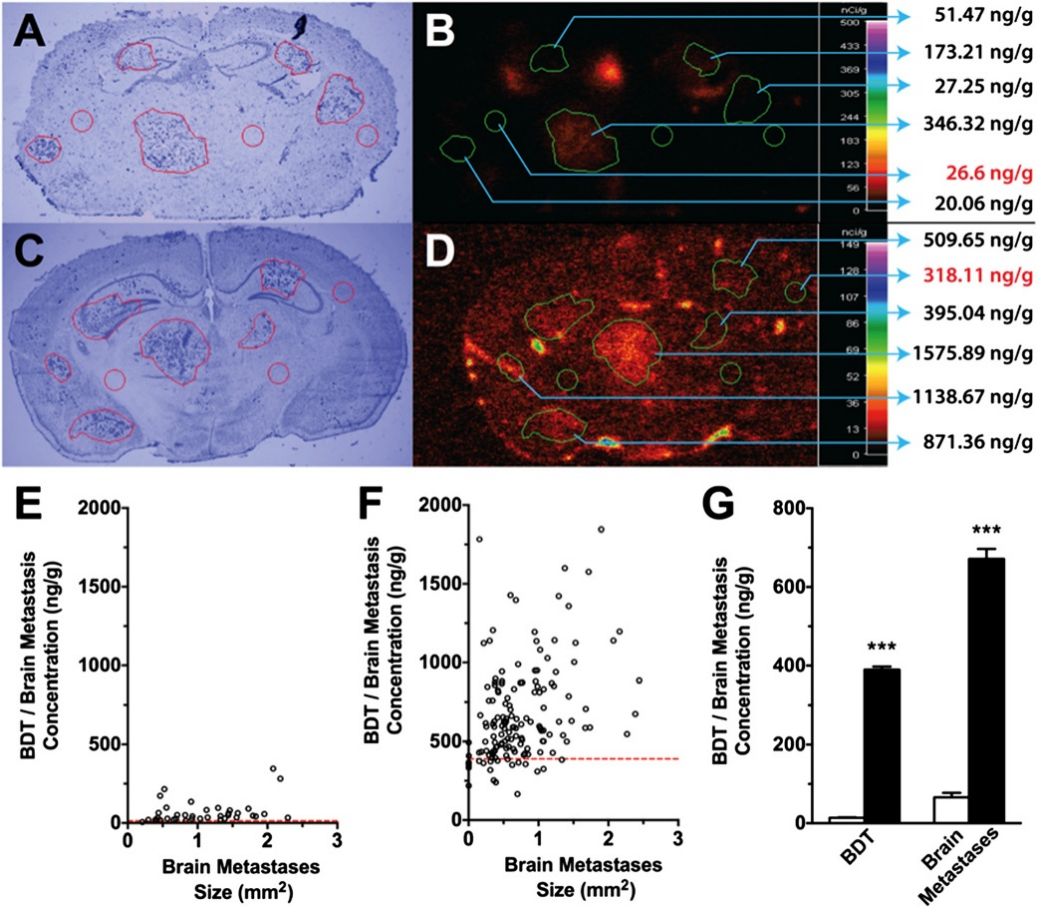
Representative image of 231Br brain metastases (**a**) and corresponding ^14^C-Irinotecan accumulation (**b**) in metastases 2 h after intravenous administration of radiolabeled irinotecan. Representative image of 231Br brain metastases (**c**) and corresponding ^14^C-NKTR-102 accumulation (**d**) in metastases 6 h after intravenous administration of radiolabeled NKTR-102. ^14^C-irinotecan concentration versus 231Br lesion size in individual metastases (**e**). ^14^C-NKTR-102 concentration versus 231Br lesion size in individual metastases (**f**). Dashed line in panel (**e**) and **f** represents mean BDT ^14^CIrinotecan and ^14^C-NKTR-102 concentration respectively. Mean BDT and lesion accumulation of ^14^C-Irinotecan (white columns) and ^14^C-NKTR-102 (black columns) (**g**). Mean lesion accumulations of ^14^C-Irinotecan and ^14^C-NKTR-102 were significantly different. All data are Mean ± SEM (*n* = 8-10)

After establishing that NKTR-102 accumulates in brain tumor tissue and serves as a reservoir for SN38, we wanted to determine if NKTR-102 accumulates in a similar fashion in BMBC. Rather than injecting tumor cells orthotopically, we injected MDA-MB-231Br cells intracardially and waited 32–35 days for mice to develop neurological symptoms before injecting either ^14^C-irinotecan or ^14^C-NKTR-102 intravenously to collect brains at respective plasma SN38 C_max_ times (2-h for conventional irinotecan, and 6-h for NKTR-102). Brains were sectioned and assessed for drug uptake using quantitative autoradiography (QAR). After administration of conventional irinotecan, radioactivity in brain varied widely between and within metastases and ranged from ~25 ng/g to ~350 ng/g (Fig. 3a, b, e), averaging 66 ng/g, 4.7 times the average radioactivity of brain distant to tumor (BDT; contralateral region) (14 ng/g). After administration of NKTR-102, irinotecan radioactivity in BM ranged from ~390 ng/g to ~1800 ng/g (672 ± 25 ng/g) (Fig. 3c, d, f), significantly higher (*p* < 0.05) compared to radioactivity after administration of conventional irinotecan (65.7 ± 11 ng/g) (Fig. 3g). Average BM radioactivity following NKTR-102 was 622 ng/g. Although only twice as high as the average radioactivity in BDT, we speculate that higher plasma NKTR-102 levels at the 6-h timepoint (720 ng/mL, Table 1) largely explains radioactivity in BDT as tracer remaining within the vasculature.

### NKTR-102 prolongs survival of animals with breast cancer brain metastases

**Fig. 4:**
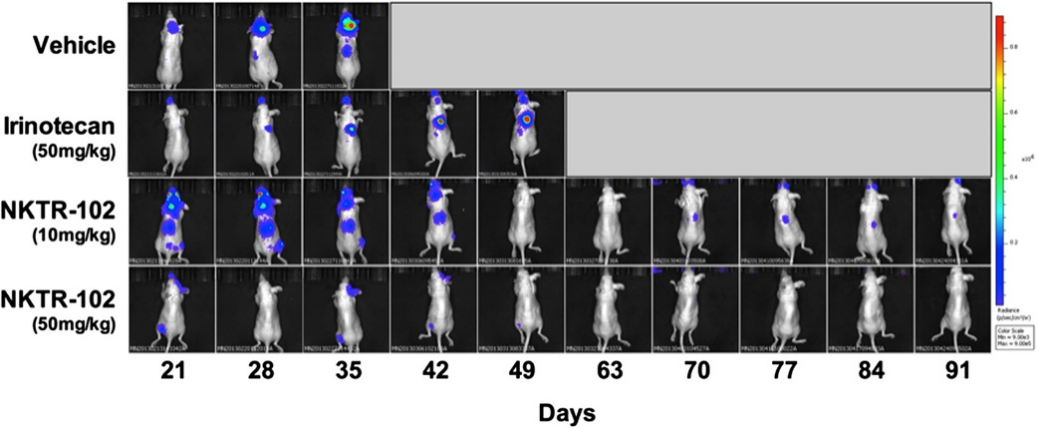
Representative bioluminescence images of mice bearing metastases and treated with either irinotecan or NKTR-102 are shown in the top row. Day 56 was omitted to conserve space

**Fig. 5:**
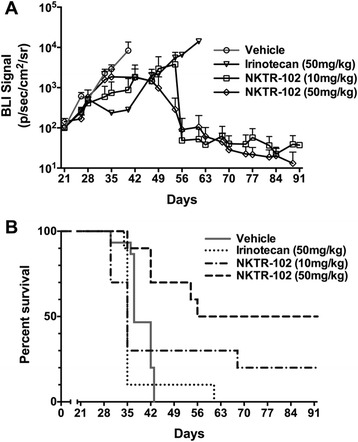
**a** Mean BLI signal versus time by treatment in mice exhibiting brain metastases. Treatment was initiated on day 21. Each data point represents mean ± SEM (*n* = 5-18 per time point). (**b**) Survival analysis of mice bearing brain metastases of human breast cancer and treated weekly via IV bolus (tail vein injection) with vehicle, irinotecan (50 mg/kg), NKTR-102 (10 mg/kg), or NKTR-102 (50 mg/kg), starting 21 days post intracardiac injection of tumor cells. Median survival time was 37 days for vehicle, 35 days for irinotecan, 35 days for NKTR-102 (10 mg/kg), and 74 days for NKTR-102 (50 mg/kg)

Having established that NKTR-102 distributes to BMBC, we then evaluated whether elevated concentrations of NKTR-102 in BM would translate to improved survival in an experimental model of BMBC. To evaluate this, we intracardially injected MDA-MB-231Br cells and allowed metastatic lesions to develop in brain. During development of BM, tumor growth was monitored using bioluminescence imaging (Fig. 4). Similar to our previous work [45], this model produced detectable and quantifiable tumor growth in the brain 21 days post injection, the day drug treatment started, emphasizing that BM formed prior to drug exposure. Animals treated with vehicle, tumor burden increased nearly 100-fold (Fig. 5a) over three weeks at which time all animals became moribund and required sacrifice, resulting in a median survival of 37 days (Fig. 5b). Weekly administration of conventional irinotecan at 50 mg/kg was unable to prolong survival; median survival was the same as observed for the vehicle group, with one animal surviving until day 60 (Fig. 5b). With regard to NKTR-102, drug was administered at two dose levels: 50 mg/kg, equivalent to the irinotecan dose administered, as well as 10 mg/kg, a dose previously demonstated to have activity in a subcutaneously implanted MX-1 breast cancer model (personal communication). Weekly administration of 50 mg/kg NKTR-102 increased median survival to 74 days, 39 days longer compared to irinotecan at an equivalent dose, and five of ten animals survived to completion of the study (Fig. 5b). Of interest, metastatic tumor burden decreased two weeks after the start of NKTR-102 treatment and was nearly eliminated in animals receiving treatment during the final two weeks of the study (Fig. 5a). Even at the 10 mg/kg dose concentration, we observed tumor burden levels decreased to approximately 50 % of irinotecan treated animals (Fig. 5a), with 3 animals surviving to study completion at 91 days (~2.5 longer than vehicle control); however, no increase in median survival was observed in this group relative to the vehicle group (Fig. 5b).

### NKTR-102 treatment decreases the number and size of brain metastasis

**Fig. 6:**
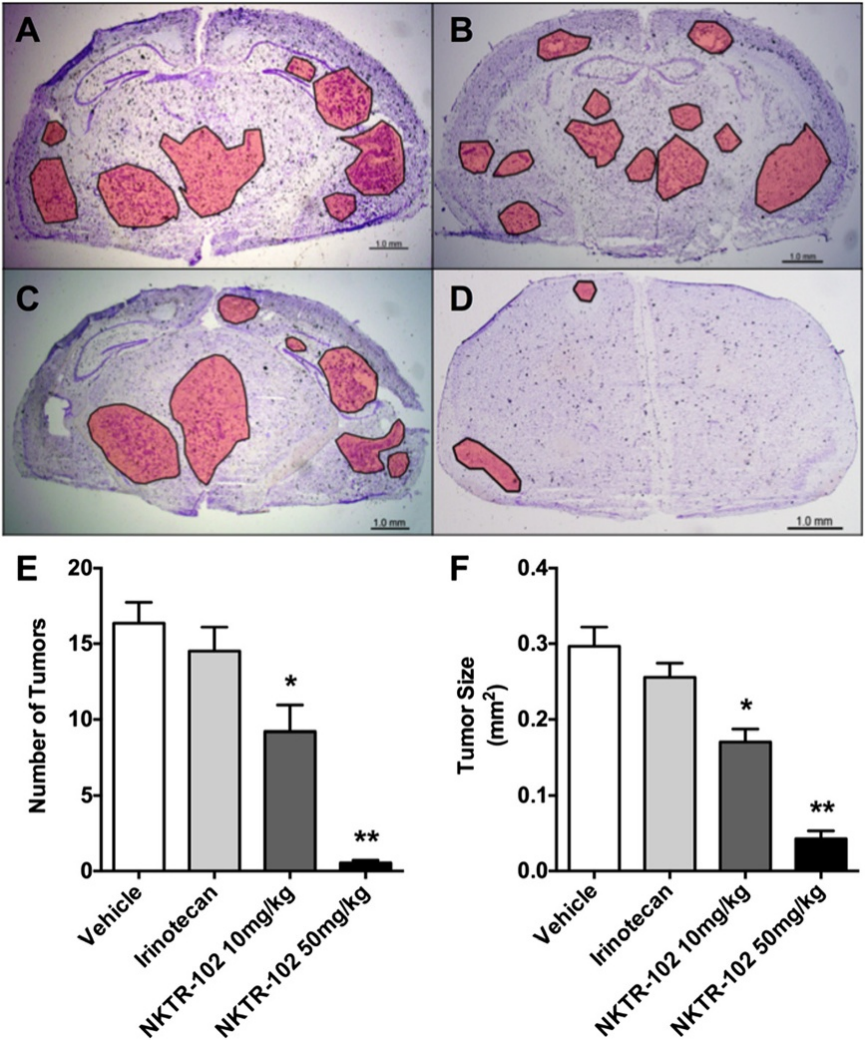
Representative cresyl-violet stained brain sections from **a** vehicle, (**b**) irinotecan, (**c**) NKTR-102 10 mg/kg, and (**d**) NKTR-102 50 mg/kg treated animals. Tumor regions are outlined and shaded. (**e**) The number of detectable brain metastases by treatment. Significant differences (*p* < 0.05 and *p* < 0.01) were observed in the number of CNS metastases in animals treated with low dose (9.2 ± 1.7) and high dose NKTR-102 (0.54 ± 0.2) compared to vehicle (16.4 ± 1.4) and irinotecan (14.5 ± 1.6) treated animals. (**f**) The average size of the CNS metastasis (μm^2^) was smaller in animals treated with low dose (0.17 ± 0.02) and high dose NKTR-102 (0.04 ± 0.01) compared to vehicle (0.29 ± 0.3) and irinotecan (0.26 ± 0.2) treated animals. All data are Mean ± SEM (*n* = 5-10)

In our last experiments, we evaluated histological characteristics of metastatic lesions (Fig. 6a–d) in brains from animals used in the survival study. We observed no significant differences (*p* > 0.05) in the number (Fig. 6e) or size (Fig. 6f) of MDA-MB-231Br lesions in brain between vehicle and irinotecan treated animals. However, animals treated with low dose NKTR-102 (10 mg/kg) exhibited a ~43 % reduction in lesions, both in metastasis number and size compared to the vehicle group. Moreover, administration of high dose NKTR-102 (50 mg/kg) reduced average BM number by ~97 % and size by ~87 %. Histological data appears to support survival study observations.

## Discussion

NKTR-102 overcomes several limitations of irinotecan therapy. Administration of irinotecan produces a SN38 plasma half-life of 24–48 h, well below a half-life of ~50-days after NKTR-102 administration resulting in continuous drug exposure between dosing cycles. The sustained exposure observed with NKTR-102 in cancer patients was associated with promising activity during both Phase 1 [37] and Phase 2 [38, 39] studies of NKTR-102. In particular, patients with third-line metastatic breast cancer of all types (including triple-negative disease) who received NKTR-102 demonstrated a confirmed objective response rate of 29 % by RECIST criteria [39]. This efficacy was achieved with manageable and significantly milder side effects than reported for irinotecan therapy, in which the most common Grade 3/4 toxicity was diarrhea, occurring in 20-23 % of patients [39]. In animal models of cancer, the polymer moiety in NKTR-102 led to prolonged circulation time and tumor localization, resulting in increased tumor exposure to SN38 that correlates well with superior suppression of tumor growth compared with irinotecan [36]. Here we show superior properties imparted by the polymer in NKTR-102 translate to advantages over irinotecan in a setting of CNS tumors. NKTR-102 crossed the BTB and preferentially accumulated in brain tumors, as evidenced by the 12- to 170-times higher tumor compared to plasma concentrations for >85 % of the dosing interval. The sustained tumor NKTR-102 concentrations through 168-h post dose, indicates slow elimination of drug from the tumor. The elevated and sustained SN38 concentrations in tumor compared to plasma after NKTR-102 administration indicates the retention of NKTR-102 within brain tumors serves as a reservoir for continued release of SN38 in the brain tumor microenvironment. In contrast, administration of irinotecan produces tumor pharmacokinetics that mirror its plasma pharmacokinetics without preferential accumulation and retention, leading to exposure holidays for 70 % of the dosing interval.

The ability of NKTR-102 to cross the BTB and accumulate in brain tumor tissue appeared to contribute to the efficacy observed in this experimental model of BMBC. NKTR-102 not only increased median survival in animals with BM, but reduced established brain metastases in 50 % of animals. Based on this data, the degree of efficacy and improved survival with NKTR-102 exceeds many conventional chemotherapeutics in this model of BMBC [46].

Brain tumor entry and distribution of molecules and formulations greater than >2.5 nm are believed to occur via the enhanced permeability and retention (EPR) effect [47, 48]. This effect describes elevated permeability as a consequence of vascular dysregulation due to the proximity of proliferating tumors or metastases [49]. In addition to enhanced permeability and drug uptake, decreased clearance mechanisms resulting from tumor interstitial spaces may contribute to prolonged drug exposure [50]. The kinetics and pharmacodynamics of NKTR-102 described in this report align with previous studies of nanoparticle agents, including liposomal formulations, polystyrene-co-maleic acid conjugated nanocarzinostatins, and albumin-bound drugs that are also thought to accumulate in tumor tissue due to the EPR effect [51]. Nanoparticle formulations similar in size to the estimated hydrodynamic volume of NKTR-102 (~2-3 nm) show clinical utility by taking advantage of the EPR effect; for example, large dextran coated iron oxide nanoparticles can be used clinically for MRI imaging of brain tumors and metastases [52]. Additionally, the long systemic circulation time of NKTR-102, relative to other nanotherapeutics should further enhance its exposure to brain metastases [53]. We previously evaluated the permeability of different sized dextrans (3 kDa to 70 kDa) in this BMBC animal model which estimated average vascular pore sizes at approximately 10 nm, though with significant variability among lesions (data not shown). Pores of this size are large enough to allow penetration of NKTR-102, while larger nanotherapeutics may encounter steric hindrance [54]. Based on the data presented here, the size of NKTR-102, its enhanced pharmacokinetic profile, and previously published data in subcutaneous tumor models, we believe NKTR-102 takes advantage of the EPR effect facilitating its penetration into brain tumors and maintaining sufficient cytotoxic SN38 concentrations, leading to the regressions observed.

The ability of NKTR-102 to avoid P-glycoprotein (P-gp) mediated efflux provides an added benefit over conventional chemotherapeutics. Consistent with human brain lesions, P-gp significantly limits solute uptake into lesions in this preclinical model [55]. Conventional irinotecan is subject to P-gp mediated efflux *in vitro* and *in vivo* [56, 57], while NKTR-102 bypasses P-gp mediated efflux, resulting in enhanced drug distribution to BM [58, 59]. Strategies to modulate efflux transporter activity using transporter inhibitors (i.e. elacridar) to enhance drug distribution have been investigated in similar preclinical models [60]; however, there is some scrutiny regarding the efficacy of drugs designed to modulate efflux transporter activity [61].

The translation of results in the non-clinical to the clinical setting is often impaired by preclinical doses that are irrelevant in the clinical setting. We elected to limit NKTR-102 doses to 50 mg/kg irinotecan equivalents to maintain plasma SN38 concentrations at ≥2 ng/mL. Similar plasma SN38 trough concentrations are achieved clinically with administration of 145 mg/m^2^ NKTR-102 given every three weeks [37]. This is the recommended dose and schedule for single agent use of NKTR-102, increasing the likelihood that the nonclinical activity described here translates to efficacy in the clinical setting. The phase 3 BEACON (Breast Cancer Outcomes With NKTR-102), NCT01492101) study in patients with advanced breast cancers allowed enrollment of patients with stable brain metastases enabling an initial assessment of whether the promising efficacy observed in this experimental mouse model of breast cancer brain metastases translates to the clinical setting.

## Conclusions

In summary, data presented herein demonstrate efficacy of NKTR-102 in an experimental mouse model of BMBC. The efficacy observed correlates with the ability of NKTR-102 to cross the BTB, leading to preferential accumulation and retention in brain tumor, followed by sustained efficacious concentrations of the active metabolite SN38. Together, these data demonstrate the potential use of NKTR-102 in patients diagnosed with BMBC.

## Additional file

Additional file 1: **Supplementary methods contains fine details of various protocols and assays used in this work.** (PDF 91 kb)

## Abbreviations

NKTR-102: Nektar etirinotecan pegol formulation
BM: Brain metastases
BMBC: Brain metastases of breast cancer
TNBC: Triple negative breast cancer
BBB: Blood–brain barrier
BTB: Blood-tumor barrier
CNS: Central nervous system
CPT-11: Camptothecin-11
5-FU: 5-fluorouracil
Top1: Topoisomerase 1
PEG: Polyethylene glycol
P-gp: P-glycoprotein
QAR: Quantitative autoradiography
BDT: Brain distant to tumor
BLI: Bioluminescence imaging
H&E: Hematoxylin and eosin
EPR: Enhanced permeability and retention
SEM: Standard error of the mean
