# Supplementary material for: NKTR-102 Efficacy versus irinotecan in a mouse model of brain metastases of breast cancer
Source: BMC Cancer. 2015 Oct 13;15:685. doi: 10.1186/s12885-015-1672-4 (PMC4604629; doi:10.1186/s12885-015-1672-4)
Supplement: Additional file 1: — Supplementary methods contains fine details of various protocols and assays used in this work. (PDF 91 kb) [file 12885_2015_1672_MOESM1_ESM.pdf]

SUPPLEMENTAL METHODS

Chemicals

All doses and plasma and tumor concentrations of NKTR-102 are expressed based on irinotecan content, enabling a direct comparison with irinotecan. Irinotecan and NKTR-102 were dissolved in vehicle containing 6 mg/mL lactic acid and 5% Dextrose in Water (4.5), and the pH was adjusted to 5-6. Dosing solutions were filtered into sterile containers via 0.45μm PVDF filters (Millipore) prior to administration.

Cell Culture

For preparation of MDA-MB-231Br-Luc cells for intracardiac injection, cells were grown to 70% confluency, trypsinized, and rinsed twice in 4°C sterile PBS to remove all traces of serum and trypsin. Cells were re-suspended in serum-free 4°C DMEM media and placed on ice.

LC/MS/MS to estimate NKTR-102 in Brain Tumors

Intracranial implantation was initiated as previously described . under 2% isoflurane and injection of 5×10^5 MDA-MB-231Br-Luc cells using a stereotaxic manual injector (Stoelting, Wood Dale, IL). Coordinates were set to 2.5 mm right (lateral) to bregma and approximately 4 mm deep. Tumors were allowed to grow to 20-30 mg in size (30 days or until neurological symptoms appeared) before intravenous bolus administration of irinotecan (50 mg/kg) or NKTR-102 (50 mg/kg). Animals (n=5/timepoint) were sacrificed under anesthesia (ketamine/xylazine; 100 mg/kg and 8 mg/kg respectively) at pre-determined time points (pre-dose, 2, 6, and 24 hours after irinotecan; pre-dose, 6, 24, 168 hours after NKTR-102) to harvest blood and tumor samples. Blood (2 mL) was collected into tubes containing NaF and Na_2EDTA and

processed to plasma (5000 xg, 5 min, 2-8 °C). Plasma was stabilized with 2 mM PMSF and 1% glacial acetic acid, and stored frozen (- 80 °C) until analysis. Brain tumors were surgically resected, weighed, added to 2 mL preservation solution (2 mM PMSF and 1% glacial acetic acid, and stored frozen (- 80 °C)) until analysis. Plasma and brain tumor samples were assayed for NKTR-102, irinotecan, and SN38 using liquid chromatography–tandem mass spectrometry (LC/MS/MS) methods. Briefly, plasma and tumor samples were defrosted; tumor samples were homogenized prior to extraction of analytes. Irinotecan and SN38 were extracted using protein precipitation with acetonitrile followed by liquid-liquid extraction with methyl tertiary butyl ether and quantified by LC/MS/MS, using calibration standards containing irinotecan and SN38. LC/MS/MS used an Onyx Monolythic C18, 100 x 3-mm column, operated at 30 °C, at a flow rate of 1-2.5 mL/min with a gradient consisting of 0.1% formic acid in water and 0.2% formic acid in 75:25 acetonitrile:methanol coupled to an API 4000 (Applied Biosystems). NKTR-102 was extracted from plasma using protein precipitation with acetonitrile. Supernatant from protein precipitation containing NKTR-102 was directly quantified by LC-MS/MS, using calibration standards consisting of NKTR-102. LC-MS/MS used an Intrada WP-RP, 50 x 2.0-mm column, operated at 60 °C, at a flow rate of 0.5-1.0 mL/min with a gradient consisting of 0.8% formic acid in water and 0.8% formic acid in acetonitrile, coupled to an API 4000 (Applied Biosystems). The lower limits of quantitation for NKTR-102 were 5 ng/mL in plasma, and 2 ng/g in brain tumor homogenate. The lower limits of quantitation for irinotecan and SN38 were 1 ng/mL and 0.2 ng/mL in plasma, and 0.05 ng/g and 0.009 ng/g in tumor homogenate, respectively.

Quantitative autoradiography

Microscope slides with brain slices were placed in QAR cassettes (FujiFilm Life Sciences, Stamford, CT) along with ^{14}C autoradiographic standards (GE Healthcare, Piscataway, NJ). A phosphor screen (FujiFilm Life Sciences, 20 × 40 super-resolution) was placed with the slides and standards and allowed to develop for 6 to 14 days. QAR phosphor screens were developed in a high-resolution phosphor-imager (FUJI FLA-7000, FujiFilm Life Sciences) and converted to digital images. Digital QAR images were calibrated to ^{14}C standards and analyzed using MCID Analysis software (InterFocus Imaging LTD, Linton, Cambridge, England).

Histologic Evaluation of Brain Metastasis after Treatment

At the end of the survival study, brains from select animals (n=4/treatment group) were harvested, sectioned, and mounted onto slides. Slides were stained with H&E to visualize brain metastases. Brain sections were evaluated using an Olympus MVX10 microscope with a 2X objective (NA=0.5) and an optical zoom of (0.63-6.3X) to determine size and number of brain metastases. Brains were harvested from animals that were euthanized on days 37-43 in the vehicle and irinotecan treatment groups, on days 37, 42, 75, and 99 in the 10 mg/kg NKTR-102 treatment group, and on days 49, 49, 99, and 99 in the 50 mg/kg NKTR-102 treatment group. Metastases (number and size) were counted on 5-10 sections per brain and averaged across sections and animals.
